# Plasma Biomarkers Modulate *APOE4* Genotype Risk in Cognitive Decline: Insights from a Large Memory Clinic Cohort

**DOI:** 10.1002/alz70856_106816

**Published:** 2026-01-08

**Authors:** Xuemei Zeng, Rebecca A Deek, Michel N Nafash, Jeremy M. Gu, Lamia Choity, Tara K Lafferty, Marissa F Farinas, Margaret A Bedison, Rocco B Mercurio, Cristy Matan, Alexandra Gogola, Julia K. Kofler, Dana L Tudorascu, C. Elizabeth Shaaban, Jennifer H Lingler, Tharick A Pascoal, William E Klunk, Victor L. Villemagne, Milos D. Ikonomovic, Sarah B Berman, Robert Sweet, Beth E. Snitz, Ann D Cohen, M. Ilyas Kamboh, Oscar L Lopez, Thomas K Karikari

**Affiliations:** ^1^ University of Pittsburgh School of Medicine, Pittsburgh, PA, USA; ^2^ University of Pittsburgh, Pittsburgh, PA, USA; ^3^ School of Public Health, University of Pittsburgh, Pittsburgh, PA, USA; ^4^ University of Pittsburgh Alzheimer's Disease Research Center (ADRC), Pittsburgh, PA, USA; ^5^ University of Pittsburgh, School of Medicine, Pittsburgh, PA, USA; ^6^ VA Pittsburgh Healthcare System, Pittsburgh, PA, USA

## Abstract

**Background:**

The apolipoprotein E (*APOE*) 4 genotype is a major genetic risk factor of late‐onset Alzheimer's disease (AD). We examined the link between *APOE4* carriage, cognitive decline and AD plasma biomarkers in a large memory clinic cohort with annual assessments spanning up to three decades.

**Method:**

Participants at the University of Pittsburgh Alzheimer's Disease Research Center (Pitt‐ADRC) underwent baseline blood collection and cognitive function assessment using the Clinical Dementia Rating (CDR) Sum of Boxes, followed by annual CDR assessments for a median follow‐up of 3.0 years (IQR 1.9‐5.9). *APOE* genotyping was determined using TaqMan assays. Plasma *p*‐tau181, *p*‐tau217, brain‐derived tau (BD‐tau), GFAP and NfL, were measured using SIMOA assays. Linear regression and Kaplan‐Meier analysis were employed for statistical inference.

**Result:**

This study included 4,073 participants (59.9% female; 90.2% non‐Hispanic White), aged 71.9 ± 9.8 years, with 2160 being non‐demented (CDR≤0.5) at baseline. *APOE4*, but not *APOE2*, carrier status was significantly associated with worse (higher) CDR scores (*p* <0.001) and higher levels of *p*‐tau181, *p*‐tau217, BD‐tau, and GFAP (all *p* < 0.001), but not NfL. The significance of these associations was not influenced by the number of *APOE4* alleles. *APOE4* carriers experienced a faster decline in cognitive function, with a median cognitive stable time of 5.0 years compared to 6.1 years for non‐carriers (log‐rank *p* <0.001). However, this *APOE4*‐dependent cognitive decline was only apparent in participants with <=median plasma biomarker concentrations but disappeared in individuals with >median biomarker levels.

**Conclusion:**

This study shows that the *APOE4* genotype exerts a significant, biomarker‐dependent risk on cognitive decline at early stages of the disease. These findings can guide personalized risk assessment and improve clinical management of AD.

